# Effect of Additional Apical Preparation on Retreatment of Curved Root Canals Filled with Different Sealers

**DOI:** 10.1055/s-0042-1750693

**Published:** 2022-09-08

**Authors:** Karina I. M. C. Tavares, Jader C. Pinto, Airton O. Santos-Junior, Marco A. H. Duarte, Juliane M. Guerreiro-Tanomaru, Mario Tanomaru-Filho

**Affiliations:** 1 Department of Restorative Dentistry, School of Dentistry, São Paulo State University, Araraquara, São Paulo, Brazil; 2 Department of Dentistry, Endodontics and Dental Materials, School of Dentistry, University of São Paulo, São Paulo, Brazil

**Keywords:** retreatment, root canal preparation, root canal filling materials, X-ray microtomography

## Abstract

**Objective** This study assessed the influence of additional apical preparation on material removal during retreatment of curved root canals filled with different sealers.

**Materials and Methods** Twelve mesial roots of mandibular molars with two separate canals and curvature between 25 and 35 degrees were selected. The working length was established 1-mm short of the apical foramen, and all the root canals were prepared using ProDesign R (PDR) 25/0.06. After preparation, the root canals were divided in two groups ( *n* = 12) and filled by continuous wave condensation technique with NeoMTA Plus or AH Plus. All the root canals were retreated using rotary ProDesign Logic RT 25/0.08, reciprocating PDR 25/0.06 and apical preparation with PDR 35/0.05. Additional apical preparation was performed with ProDesign Logic (PDL) 50/0.01. The samples were scanned using a Skycan 1176 micro–computed tomography (micro-CT), voxel size 8.74 µm, before and after the retreatment procedures. Percentages of remaining filling material were evaluated.

**Statistical Analysis** The data were submitted to paired and unpaired *t* -tests ( *α* = 0.05).

**Results** Percentage of remaining filling material was similar between the root canals filled with NeoMTA Plus or AH Plus sealer after retreatment using PDR 35/0.05, and after additional apical preparation using PDL 50/0.01 ( *p* > 0.05). However, PDL 50/0.01 significantly decreased the percentage of remaining filling material in the apical third after the additional apical preparation for both sealers ( *p* < 0.05).

**Conclusion** NeoMTA Plus or AH Plus did not influence the retreatment of curved root canals. ProDesign Logic 50/0.01 promoted greater filling material removal in the apical third.

## Introduction

Endodontic retreatment aims to remove the filling material allowing cleaning and disinfection of the root canal system. 1 However, the presence of root canals with curvature makes it difficult to remove the filling material from the apical third in the root canal, contributing to the failure of endodontic retreatment. 2 3

Different endodontic sealers associated to gutta-percha are usually used for filling root canals. 4 Sealers based on calcium silicate may be more difficult to remove during retreatment 5 6 in comparison to resin-based sealers. Furthermore, obturation techniques influence the removal of the remaining filling material in curved root canals. 6 Thermoplastic obturation techniques provide a homogeneous filling, 7 8 making it difficult to remove the filling material. 6 Retreatment of curved root canals filled by the single-cone technique presented similar removal of NeoMTA Plus (NuSmile, Houston, Texas, United States) or AH Plus (Dentsply DeTrey GmbH, Konstanz, Germany). 9 However, no studies using continuous wave condensation technique with different sealers evaluated the retreatment in curved root canals.

No retreatment technique using different instruments is able to completely remove the remaining filling material, 10 especially in the apical third. 3 Thus, additional apical preparation using nickel–titanium (NiTi) instruments with large diameter and small taper has been proposed to improve the removal of remaining material in curved canals. 11 12 ProDesign Logic 50/0.01 (Easy Equipamentos Odontológicas, Belo Horizonte, Minas Gerais, Brazil) has diameter of 50 and a small taper of 0.01. This instrument significantly reduces the amount of filling material in the apical third of maxillary lateral incisors 11 and curved canals of mandibular molars. 12 However, no study evaluated this procedure in the retreatment using different sealers.

This study evaluated the percentage of remaining filling material in curved root canals filled with NeoMTA Plus or AH Plus after retreatment with ProDesign R and the effectiveness of the additional apical preparation using ProDesign Logic 50/0.01. The first null hypothesis was that there would be no difference in removing filling material in root canals filled with NeoMTA Plus or AH Plus. The second null hypothesis was that the ProDesign Logic 50/0.01 instrument would not influence the percentage of remaining filling material in curved canals.

## Materials and Methods

### Sample Size Calculation

G* Power 3.1.7 for Windows program (Heinrich-Heine-Universitat Dusseldorf, Dusseldorf, Germany) was used for the sample size calculation. The *t* -test was used with an alpha-type error of 0.05 and beta power of 0.90. Previous study was used to determine the effect size of 1.389. 9 A total of 12 specimens were indicated as being the ideal size required.

### Specimen Selection

All procedures were approved by the University Ethics Committee (Protocol CAAE No. 64736116.4.0000.5416). Mandibular first and second molars stored in 0.1% thymol solution at 5°C were used. The inclusion criteria were two independent mesial root canals, type IV, according to configuration to the classification of Vertucci, 13 angle of curvature between 25 and 35 degrees by the method of Schneider, 14 and radius of curvature smaller than 10 mm, 15 besides complete apical formation, absence of root fractures, calcifications, or internal resorptions. For this purpose, the specimens were selected by a digital radiography system (Kodak RVG 6100 Digital Radiography System, Marne-la-Vallée, France) and micro–computed tomography (micro-CT SkyScan 1176; Bruker-micro-CT, Kontich, Belgium). The first scanning was performed at a low-resolution (isotropic voxel 35 µm) under the following settings: copper and aluminum filter, exposure time of 87 ms, frame averaging 3, rotation angle 180 degrees, rotation step of 0.5, 80 kV X-ray tube voltages, and 300 µA anode current. After the exclusion of all teeth that did not reach the inclusion criteria, 12 specimens were selected. The specimens were embedded in condensation silicone (Oranwash, Zhermack SpA, Badia Polesine, Italy) to simulate the periodontal ligament.

### Root Canal Preparation

Conventional access cavities were performed, and the root canals were explored by using a size no. 10 K-file (Dentsply Sirona, Ballaigues, Switzerland). The working length (WL) was established 1-mm short from the apical foramen. All root canals were prepared with ProDesign R (PDR; Easy Equipamentos Odontológicos, Belo Horizonte, Minas Gerais, Brazil) 25/0.06, operated by electric motor VDW SILVER (VDW GmbH, Munich, Germany) in “RECIPROC ALL” mode with in-and-out movements up to the WL. Root canals were irrigated with 5 mL of 2.5% sodium hypochlorite (NaOCl; Ciclo Farma, Serrana, São Paulo, Brazil) using a 5-mL syringe (Ultradent Products Inc., Salt Lake City, Utah, United States) and a Navitip 30-G needle (Ultradent Products Inc.) at 2-mm short of the WL. Final irrigation was performed using 5 mL of 2.5% NaOCL, 2.5 mL of 17% EDTA (Biodinâmica, Ibiporã, Paraná, Brazil) under agitation for 3 minutes with a gutta-percha cone size 25/0.06, followed by irrigation with 5 mL of distilled water.

### Root Canal Filling

After preparation, the root canals were divided into two experimental groups ( *n* = 12), using stratified random sampling, considering the volume of prepared canals. The root canals were filled with NeoMTA Plus or AH Plus ( Table 1 ) by the continuous wave of condensation technique—Thermo Pack II (Easy Equipamentos Odontológicos, Belo Horizonte, Minas Gerais, Brazil).

**Table 1:** Materials, their manufacturer, composition, and proportions used

Materials	Manufacturer	Composition	Proportion
NeoMTA Plus	NuSmile, Houston, Texas, United States	Powder: tricalcium silicate, dicalcium silicate, tantalum oxide, tricalcium aluminate, and calcium sulfate. Liquid/gel: water-based gel with thickening agent and water-soluble polymers	0.33 g powder:150 μL liquid/gel
AH Plus	Dentsply DeTrey GmbH, Konstanz, Germany	Bisphenol-A epoxy resin, bisphenol-F epoxy resin, calcium tungstate, zirconium oxide, silica, iron oxide pigments dibenzyl-diamine, aminoadamantane, silicone oil	1 g:1 g (paste/paste)

Gutta-percha cones 25/0.06 (Tanariman, Manacapuru, Amazonas, Brazil) were selected according to the tip size and taper measured in the profilometer (Profile Projector Nikon Model 6C-2; Nikon, Tokyo, Japan). After the radiographic evaluation of the gutta-percha cone selected, the sealer was placed into root canal using a Lentulo 25 instrument (Dentsply Sirona) and manual K-file 25 pre-curved (Dentsply Sirona). After this, the gutta-percha cone covered by one of the endodontic sealers was placed in the root canal. The thermoplastic plugger from Termo Pack II System was used to plasticize and cut the gutta-percha. The compaction of the gutta-percha was performed using NiTi condensers (Easy Dental Equipamentos, Belo Horizonte, Minas Gerais, Brazil) within the apical root canal up to 3 mm of the working length. After this, the thermal gutta-percha injector was used to inject the warmed gutta-percha. Compaction was performed with NiTi condensers (Easy Dental Equipamentos, Belo Horizonte, Minas Gerais, Brazil). A radiograph was taken in the buccal-lingual and mesiodistal to verify the quality of the obturation.

After filling the root canals, each sample was sealed with provisional Coltosol (Vigodent, Rio de Janeiro, Rio de Janeiro, Brazil) restorative material and kept in an oven at 37°C, in 95% humidity, for 7 days to allow the sealers to set completely.

### Root Canal Retreatment

After removing the temporary restorations, all root canals were retreated with ProDesign Logic RT (PDL RT; Easy Equipamentos Odontológicos, Belo Horizonte, Minas Gerais, Brazil) and PDR systems, operated on an electric motor VDW SILVER. PDL RT 25/0.08 instrument was operated in rotary motion at 600 rpm and torque 3 ncm, with in-and-out movements to remove the cervical and middle third filling. PDR 25/0.06 instruments were activated in “RECIPROC ALL” mode, with in-and-out movements, up to WL to remove apical third filling. The root canals were enlarged with PDR 35/0.05 as described above. Each root canal was irrigated with 6 mL of 2.5% NaOCl (2 mL for each instrument). Final irrigation was performed, as described in the preparation step.

### Additional Apical Preparation

Following the retreatment procedures, an apical enlargement was performed with ProDesign Logic (PDL) 50/0.01. The instrument was operated on an electric motor VDW SILVER, in rotary motion, at a speed of 350 rpm and torque 1 ncm, with in-and-out movements up to the WL. The root canals were irrigated with 2 mL of 2.5% NaOCl. Final irrigation was performed as previously described.

### Micro–Computed Tomography Scanning and Analysis

The roots were scanned using SkyScan 1176 micro-CT before and after retreatment procedures. The roots were positioned in a standardization device, allowing the specimens to remain in the same position during scanning procedures. The scanning parameters were copper and aluminum filter, 90 kV X-ray tube voltages, 278 µA anode current, exposure time of 125 ms, rotation angle of 180 degrees, rotation step of 0.5, frame averaging 4, and isotropic voxel of 8.74 µm. The images obtained were reconstructed using the NRecon software (v.1.6.3; Bruker-micro-CT) and were superimposed using geometric alignment in the Data Viewer software (v.1.5.1; Bruker-micro-CT). Volumetric analysis was performed in all root canals using CTAn software (v.1.14.4; Bruker-micro-CT) with specific task lists. Root canal volume and remaining filling material were quantified after retreatment steps. The grayscale range required to recognize each object under study was determined in a density histogram using the adaptive threshold method. The following formula was used to obtain the percentage remaining filling material: percentage remaining filling material = (filling material after retreatment × 100 / filling material before retreatment). The evaluation was performed in the apical third and cervical/middle thirds of the root canals. The value of approximately 9 mm was determined for the total length analysis and approximately 3 mm for the apical third and 6 mm for cervical/middle thirds. Representative images were performed using CTVox software (v.3.2; Bruker-micro-CT).

### Statistical Analysis

The data were submitted to the Shapiro–Wilk normality test. The no-paired *t* -test was used to compare groups. The paired *t* -test was used to compare the root canals retreatment before and after additional apical preparation with PDL 50/0.01. The level of significance was 5% for all the analyses.

## Results

**Fig. 1:**
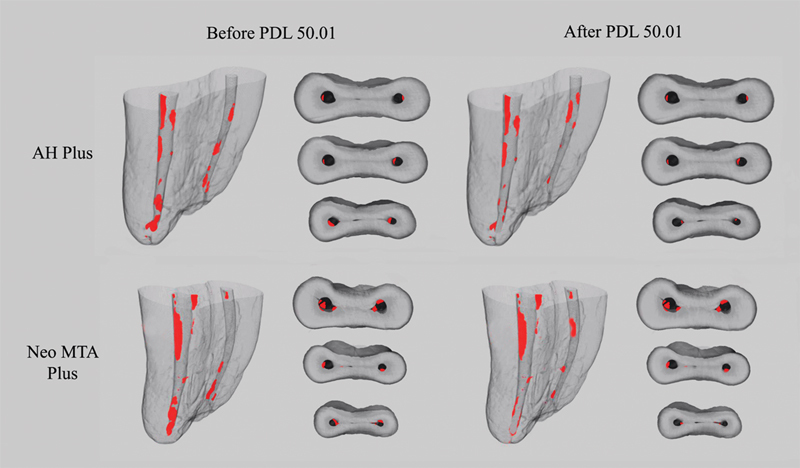
Three-dimensional reconstructions and cross-sectional images of mesial root canals of mandibular molars after the steps of retreatment and additional apical preparation with ProDesign Logic (PDL) 50/0.01. Remaining filling material is showed in red.

The percentage of remaining filling material was similar between the root canals filled with AH Plus or NeoMTA Plus sealer after the retreatment procedure with PDR 35/0.05 and after additional apical preparation with PDL 50/0.01 ( *p* > 0.05; Table 2 ). PDL 50/0.01 significantly decreased the percentage of remaining filling material in the apical third after the additional apical preparation ( *p* < 0.05; Table 2 ; Fig. 1 ).

**Table 2:** Means and standard deviations of the percentage of remaining filling material before and after additional apical preparation with ProDesign Logic 50/0.01 in curved root canals filled by continuous wave of condensation with NeoMTA Plus or AH Plus sealers

		Before PDL 50/0.01	After PDL 50/0.01	Reduction in residual filling material (%)
NeoMTA Plus	Cervical/middle	15.98 ± 4.76 a	14.05 ± 5.98 a	8.43 ± 1.87
	Apical	13.04 ± 8.06 a	7.24 ± 5.50 b	48.76 ± 7.31
AH Plus	Cervical/middle	14.56 ± 5.28 a	13.76 ± 4.27 a	8.04 ± 2.34
	Apical	19.83 ± 9.34 a	8.63 ± 3.38 b	54.87 ± 8.21

Note: The percentage of reduction in residual filling material was also described.

There was no statistical difference between the groups ( *p* > 0.05).

a,b Values represent significant differences intragroup ( *p* < 0.05).

## Discussion

The percentage of remaining filling material in the cervical/middle and apical thirds was similar between the root canals filled with NeoMTA Plus and AH Plus. Thus, the first null hypothesis was accepted. A previous study showed similar result when comparing the retreatment between NeoMTA Plus and AH Plus. 9 These results can be attributed to the mechanical and physical properties of the evaluated sealers. NeoMTA Plus presents small particles 16 and ability to penetrate into dentinal tubules. 17 In addition, the higher bond strength of NeoMTA Plus when compared with Biodentine (Septodont, Saint-Maur-des-Fosses, France) and White ProRoot MTA (Dentsply Tulsa Dental, Oklahoma, United States) and the higher resistance to dislodgment of AH Plus than MTA Fillapex (Angelus, Londrina, Paraná, Brazil) and EndoSequence BC Sealer (Brasseler, Savannah, Georgia, United States) 16 18 may favor their interaction with the dentin surface for both sealers.

Several mechanized systems with different kinematics have been proposed for endodontic retreatments. 10 Rotary motion increases the gutta-percha removal to the cervical direction, reducing the compaction of filling material in the apical third. 19 On the other hand, the reciprocating motion improves the torsional and cyclic fatigue resistance of the instruments, 20 allowing safer apical preparation of curved root canals, and filling removal similar to the rotary kinematics. 10 Therefore, the aforementioned statements justify the use of a PDL RT 25/0.08 in the cervical and middle thirds, and PDR 25/0.06 in the apical third for the retreatment's procedures in the present study. Subsequently PDR 35/0.05 reciprocating file was used, since apical enlargement larger than the initial root canal preparation is indicated to improve filling material removal 9 10 and does not influence in the postoperative pain. 21 22 However, a considerable amount of remaining filling material could be observed in all root canals since rotary and reciprocation instruments cannot completely remove the filling material. 10

The apical third is considered a critical zone due to the difficulty of cleaning and disinfection. 23 Therefore, the apical enlargement allows greater contact of the instrument and dentin walls reducing debris, providing better effect of the irrigating solution 24 and root canal filling. 25 In addition, the decrease of residual filling material was observed after apical enlargement from size 25 to 40, 26 with a success rate of 90.4% after 2 years. 27 Although our study showed a percentage of remaining material less than 20% in the apical third after apical enlargement, the complete removal of filling material was not possible.

Additional apical preparation using in the retreatment are encouraged since previous studies observed better filling removal after retreatment in curved canals. 9 12 In our study, PDL 50/0.01 decreased the percentage of remaining filling material in the apical third for both sealers. Thus, the second hypothesis was partially rejected. Similar result was observed when PDL 50/0.01 was used in curved root canals filled with AH Plus. 12 In addition, the small and constant taper 0.01 avoided unnecessary dentin removal in the cervical/middle thirds, preventing root weakening 26 and favoring less material extrusion. 28 In agreement, previous investigation showed the safety of this file during retreatments of curved canals since PDL 50/0.01 has high flexural strength and provides apical enlargement with no root canal transportation. 12

Curved root canals may favor the occurrence of apical transportation, zipping or crack formation during preparation, and retreatment procedures, with impair the treatment outcome. 2 29 Mesial root canals of mandibular molars with a curvature of 25 and 35 degrees were used in the present study representing moderate root curvatures. 30 Scanning in micro-CT using lower resolution (35 µm) has been performed to the initial selection of samples. 8 It is important to highlight that the sample distribution was performed by simple stratified randomization considering the preparation volume of the root canals. A systematic review reported that few studies performed adequate randomization of samples. 31

ProDesign Logic 50/0.01 appeared to be effective in removing the remaining material filling in the apical third in curved root canals. The use of strategies, such as additional apical preparation, can influence the success of endodontic treatment.

## Conclusion

Within the limitations of the present study, it can be concluded that NeoMTA Plus or AH Plus did not influence the retreatment of curved root canals. ProDesign Logic 50/0.01 promoted greater remaining filling material removal in the apical third.
